# Does the anatomical region predict blood loss or neurological deficits in embolized renal cancer spine metastases? A single-center experience with 31 patients

**DOI:** 10.1186/s12957-022-02676-1

**Published:** 2022-06-16

**Authors:** Anna Voelker, Georg Osterhoff, Stefanie Einhorn, Sebastian Ebel, Christoph-Eckhard Heyde, Philipp Pieroh

**Affiliations:** 1 Department of Orthopedics, Trauma and Plastic Surgery, University Hospital Leipzig, Liebigstraße 20, 04103 Leipzig, Germany; 2 Department of Radiology, University Hospital Leipzig, Liebigstraße 20, 04103 Leipzig, Germany

**Keywords:** Renal cell carcinoma, Tumor embolization, Spine surgery of metastasis, Hyper vascular spinal metastasis

## Abstract

**Background:**

No comparison of a single hypervascular tumor entity in terms of major complications in different spinal regions has been performed. We aimed to evaluate post-embolic and post-operative outcomes in anatomic regions with renal cell carcinoma (RCC) metastases to the spine.

**Methods:**

We retrospectively evaluated data from patients with confirmed, embolized, and surgically treated RCC spine metastases at a single-spine center between 2010 and 2020. Patients were divided into thoracic (TSM) and lumbar (LSM) spine metastasis groups.

**Results:**

Seventeen patients had TSM and 14 had LSM. In all cases, embolization was performed preoperatively. The ΔHb value did not differ between the two groups pre- and postoperatively (*p*=0.3934). There was no significant difference in intraoperative blood loss between both groups either within 1 day or 2 days after embolization. Neurological deficits occurred in eight patients after embolization or surgery, with no significant difference between TSM (*n*=5) and LSM (*n*=3).

**Conclusions:**

Embolization is the standard procedure for the preoperative treatment of hypervascular spinal metastases, possible up to 48 h before surgery, without the risk of higher intraoperative blood loss. Regardless of intraoperative complications, major complications can occur up to several hours after embolization. We recommend surgery the day after embolization to reliably detect neurologic complications from this procedure.

## Background

One third of patients with renal cell carcinoma (RCC) suffer from osseous metastasis. Of these, the vertebral column is affected in the majority [1]. During decompression and stabilization to achieve stability and prevent neurological deficits, the high vascularity of this tumor entity should be considered, especially regarding blood loss. Arterial embolization is a common preoperative procedure that is highly effective for different tumor entities [2, 3]. It is noteworthy that even RCC types differ in sites of metastasis and survival, probably because different tumor entities might differ in their vascularity and behavior during embolization [4].

Only a few studies have described the effect of embolization on the reduction of intraoperative blood loss in a selected patient population with spinal metastases from renal cell carcinoma [5–7].

Complications following embolization range from minor (e.g., hematoma at the injection site, atrial fibrillation, pleural exudate) to severe complications such as the rarely reported neurologic deficits due to spinal ischemia, femoral artery thrombosis, or cardiac events [8, 9].

The blood supply to the spinal cord differs anatomically in the thoracic and lumbar spine. The spinal branches are divided into anterior and posterior radicular branches. Few of the anterior radicular branches supply the anterior 2/3 of the spinal cord through the anterior spinal artery (ASA). The ASA runs continuously along the entire spinal cord and has both anterograde and retrograde blood flow. The posterior radicular arteries supply the posterior 1/3 of the spinal cord. The unique nature of the thoracic spinal region is that there are fewer radiculomedullary arteries. There were fewer collaterals and no connection between the anterior and posterior regions. The ASA is supplied mainly by the Adamkiewicz artery (AA), which is often located at the level of Th9-12 on the left side. The caliber of the ASA is often reduced in the AA region. These aspects may lead to a higher susceptibility to ischemic insults in the thoracic spinal cord. In contrast, ligatures of the lumbar spinal arteries rarely result in neurological deficits. It does not occur in the region of the conus. There is a distinct collateral plexus in the region of the conus that is supplied primarily by the lumbar radicular arteries and not by the ASA [10].

Studies on the optimal timing of surgery after embolization are controversial, and thus, a specific standard is not described. Majority of surgeries are performed within 48 h after embolization, but periods longer than 48 h have also been reported [11, 12]. Besides embolization, a previously performed chemotherapy might also reduce blood loss [13].

This study primarily aimed to assess the relationship between the anatomic region of the spine and the occurrence of new neurological deficits and blood loss during surgery following embolization. Second, the impact of the time between embolization and new neurological deficits was determined. Third, the effect of chemotherapy and the invasiveness of surgery according to the surgical invasiveness index (SII) were investigated.

## Methods

The Ethics Committee of the Medical Faculty of the University of Leipzig, Germany (037/21-ek) approved the present study. Informed consent has been obtained by the hospital contract of treatment and procedures followed were in accordance with the ethical standards of the responsible committee on human experimentation (institutional or regional) and with the Helsinki Declaration of 1975, as revised in 1983.

### Patient identification—inclusion/exclusion criteria

We retrospectively identified all patients with RCC and embolized surgically treated thoracic and lumbar spinal metastases between 2010 and 2020 admitted to a level one spine center.

The following inclusion criteria were defined: histopathological evidence of renal cell carcinoma within the spine, required surgical therapy due to a (potentially) unstable metastasis and/or compression of neurological structures by the metastasis, and embolization of the tumor feeder prior to surgery. Excluded were all patients with a metastasis to the spine with unclear histopathologic findings or a tumor entity other than RCC on histopathologic examination.

### Patient-specific parameters

The following data were collected: sex, age, first diagnosis of RCC during hospital stay, chemotherapy in the patient’s history, and length of hospital stay. The surgical indication for each metastasis with or without neural compression was based on the spinal instability neoplastic score (SINS score).

Surgical therapy included dorsal stabilization using a rod-and-screw system in all 31 patients. Patients were allocated to the thoracic spinal metastasis (TSM) and lumbar spinal metastasis (LSM) groups.

*New-onset neurologic deficits were divided into motor, sensory deficits, motor and sensory deficits specific to one nerve root, incomplete paraparesis at the time of admission, after embolization, and after surgery.*

The criteria for embolization included the presence of significant tumor vascularity on preoperative computed tomography (CT). Embolization was performed in a dedicated angiography suite. Through a femoral access, the intercostal or lumbar arteries were intubated using different guiding catheters and microcatheters. Before embolization, the obtain angiograms were checked thoroughly for critical vessels like the AA. In the absence of the AA, embolization was performed using particles (700–1000μm) and coils. Four patients were identified in whom the AA originated from the tumor-supplying intercostal artery or lumbar artery (one patient Th10 left side, one patient Th11 left side, two patients L1 left side). Here, only coils in the proximal lumbar or intercostal arteries were used.

Afterwards, the puncture site was sealed using a closure device. Surgical therapy was evaluated according to the approaches used: dorsal approach (with or without decompression of the neurological structures) and dorso-ventral approach (one- or two-stage procedure). Surgical treatment was also analyzed according to the SII [14].

Blood loss during surgery (ml) and intraoperative blood transfusion required in units were analyzed and compared between the TSM and LSM groups. Hemoglobin (Hb) values in mmol/l were evaluated preoperatively and directly postoperatively. Hb loss (ΔHb) was calculated as the difference between preoperative and postoperative Hb levels. In two-stage procedures, Hb values and blood loss were analyzed only during the primary surgery.

For further subgroup analysis, patient data from the TSM and LSM groups were divided into two groups according to the time interval between embolization and surgery. Time intervals of 24 h and 24–48 h between embolization and surgery were evaluated. The recurrence of neurological deficits and intraoperative blood loss were analyzed in both subgroups.

### Statistical analysis

Graphs and analyses were generated using GraphPad Prism software 9 (GraphPad Software, La Jolla, USA). Data are presented as mean ± standard deviation. Non-parametric data were tested for a Gaussian distribution using the Kolmogorov test. For Gaussian distributed data, an unpaired *t* test was used. Non-Gaussian distributed data were analyzed using the Mann-Whitney *U* test. Categorical data were evaluated using the Fisher’s exact test. The significance level was set at *p* < 0.05, for all tests.

## Results

Of the 31 consecutive patients, 17 had TSM and 14 had LSM without significant differences in sex, age, first tumor diagnosis at admission, previous cancer treatment, or length of hospital stay between both groups (Table 1).

**Table 1:** Descriptive data of all investigated patients with spinal metastasis of renal cancer

	Thoracic spine (TSM)	Lumbar spine (LSM)	*p* value
No. of patients	17	14	
Gender (ratio male to female)	14:3	10:4	0.6705
Age (years: mean ± standard deviation)	68.43 ±10.76	66.88 ±9.647	0.5971
First diagnosis (yes/no)	12:5	10:4	>0.9999
Previous drug cancer treatment	6:12	3:11	0.6942
Length of hospital stay (days: mean± standard deviation)	28.12 ± 15.96	29.79 ± 19.59	0.6313
Metastasis in total (*n*)	23	22	

### Surgical therapy

Dorsal stabilization was performed in all 31 patients. Decompression was performed as a laminectomy in 30 patients. In one case, no decompression was done because there was no compression of the neural structures. In this case, kyphoplasty was performed in the region of the lumbar metastatic vertebral body. In the TSM group, two patients underwent dorsoventral surgery during the same procedure. Similarly, two patients in the LSM group underwent dorsoventral surgery during a two-stage procedure. All patients in the TSM group were instrumented above and below the unstable metastasis with screws in the adjacent vertebral bodies. In the lumbar spine, the extent of instrumentation varied depending on the existing instability and decision of the surgeon with instrumentation of one or multiple adjacent vertebral bodies in each case.

### Intraoperative blood loss

The mean intraoperative blood loss was 652 ± 484 ml in all patients; however, no blood loss was documented in three patients. In both groups, the intraoperative blood loss varied from <250 to 1500 ml. In the TSM group, blood loss of more than 1500 ml occurred in two patients (1500–2000 ml, *n*=1; > 2000 ml, *n*=1). More than 1000 ml intraoperative blood loss occurred in 12 patients (TSM, *n*=6; LSM, *n*=6). Less than 1000 ml intraoperative blood loss was detected in 18 patients (TSM, *n* =11; LSM, *n*=8). Eleven blood units were administered intraoperatively in 11 patients (TSM, *n*=7; LSM, *n*=4) without significance (*p*= 0.7167). Less than or equal to three units of blood were transfused in six patients in the TSM group and in three patients in the LSM group. More than three units of the blood were transfused in one patient in the TSM as well as the LSM group.

A significant drop in Hb occurred in both groups after surgery (TSM group: mean Hb preoperatively 7.27 mmol/l, postoperatively 5.39 mmol/l, *p*<0.001; LSM group: mean preoperatively 7.08 mmol/l, postoperatively 5.48 mmol/l, *p*<0.0016). ΔHb did not differ between groups (Table 2, *p*=0.3934).

**Table 2:** Intraoperative blood loss

	Group	Mean	Std	*p* value
dHB	TSM	2.0	±0.8485	*p*=0.3934
	LSM	1.6	±0.2268	
	TSM+ch	1.9	±1.14	*p*=0.1429
	LSM+ch	0.9	±0.3464	
	TSM-ch + LSM-ch	1.831	±1.167	*p*=0.5704
	TSM+ch + LSM+ch	1.5667	±1.045	
Hb preoperatively	TSM-ch	7.218	±1.11	*p*=0.7517
	TSM+ch	7.367	±1.9	
	LSM-ch	7.209	±1.429	*p*=0.5852
	LSM+ch	6.60	±1.179	
	TSM-ch + LSM-ch	7.214	±1.249	*p*=0.5415
	TSM+ch + LSM+ ch	7.111	±1.676	
Hb postoperatively	TSM	5.345	±0.7421	*p*=0.7862
	TSMch	5.467	±1.372	
	LSM	5.418	±0.9411	*p*=0.5824
	LSMch	5.7	±0.9539	
	TSM+LSM	5.382	±0.8279	*p*=0.4564
	TSMch+LSMch	5.544	±1.045	

Intraoperative blood loss was determined based on the preoperative and postoperative hemoglobin (Hb) levels. Presented as delta (d) Hb and the absolute Hb values in the whole patient collective (TSM, LSM) and in comparison, between the patient groups without (TSM-ch, LSM-ch) and with chemotherapy (TSM+ch, LSM+ch)

### Neurology

At hospital admission, 16 patients (TSM, *n*=8; LSM, *n*=8) suffered from neurological deficits due to compression of the RCC metastasis. Five patients from the TSM group (29.41%) and three patients from the LSM group (7.14%) presented with new neurological deficits after the intervention (embolization and/or surgery). There was no significant difference between the two groups (*p*=0.698). New neurological deficits after embolization occurred in three patients (TSM, *n*=2; LSM, *n*=1). After surgical therapy, neurological deficits occurred in four patients (TSM, *n*=2; LSM, *n*=2). One patient in the TSM group who underwent surgery immediately after embolization had a new neurological deficit postoperatively. A detailed list of the types of neurology is presented in Tables 3 and 4.

**Table 3:** Occurrence of neurological deficits (ND) at admission and new occurrence of neurology during hospital stay

TSM =17 patients, LSM =14 patients	ND at admission		New ND after embolization		New ND after surgery		New ND after embolization direct surgery	
	TSM	LSM	TSM	LSM	TSM	LSM	TSM	LSM
Neurology in total (*n*)	**8** (47.06%)	**8** (57.14%)	**2** (11.77%)	**1** (7.12%)	**2** (11.77%)	**2** (14.29%)	**1** (5.88%)	**0**
Motor deficits (*n*)	2	0	1	0	0	1	0	0
Sensory deficits (*n*)	6	7	1	1	0	1	0	0
Sensory and motor deficits Specific to one nerve root (*n*)	1	1	0	0	0	0	0	0
Incomplete paraparesis (*n*)	0	0	0	0	2	0	1	0
Complete paraparesis (*n*)	0	0	0	0	0	0	0	0

The number of neurological findings classified by the TSM and LSM is shown. Subgroups were evaluated according to neurology on admission, new neurology after embolization, new neurology after surgery, and new neurology after the patient was driven from embolization directly to surgery and operated. Neurological deficits were divided into motor and sensory deficits, and incomplete and complete paraparesis

**Table 4:** Detailed medical characteristics of all patients with new neurology after embolization or surgery

Patient no	TSM or LSM	Time between embolization and surgery	Timepoint new ND	Neurological dysfunction	Blood loos ml	Blood unit n	Surgical procedure
1	Th3, Th4	<24h	Post-surgery (directly OR)	Incomplete paraparesis (ASIA C)	<1250	2	Dorsal: stabilization, decompression, cage
2	Th10, Th12	>24h <48h	Post-embolization	Sensory	<250	1	Dorsal: stabilization, decompression
3	Th10	<24h	Post-surgery	Incomplete paraparesis (ASIA C)	<500	0	Dorsal: stabilization, decompression
4	Th11	>24h<48h	Post-surgery	Incomplete paraparesis (ASIA D)	<250	0	Dorsal: stabilization, decompression
5	Th11, Th12	<24h	Post-embolization	Motoric	<500	0	Dorsal: stabilization, decompression
6	L3, L4, L5	<24h	Post-surgery	Motoric	<1250	0	Dorsal: stabilization, decompression
7	L4	<24h	Post-surgery	Sensory	<1500	1	Dorsal: stabilization, decompression; ventral: cage
8	L4, L5	>24h<48h	Post-embolization	Sensory	<500	0	Dorsal: stabilization, decompression

Detailed information of all patients who developed new neurological deficits after embolization or surgical treatment for spinal renal cell metastasis. Described is the location of the instable metastasis, time between embolization, and surgery (within 24h or within 24–48h between embolization and surgery) and time point of onset of new neurological deficit (either immediately after embolization, immediately after surgery or after surgery in case of immediate surgical treatment after embolization). Neurological deficits are described as sensory, motor, or incomplete or complete paraparesis according to the ASIA classification. Intraoperative blood loss is categorized into 250-ml steps, and blood products are reported as blood units. The surgical procedure was shown as a dorsal or ventral approach

### Embolization

Metastases were diagnosed in 45 vertebral bodies (thoracic, *n* = 23; lumbar, *n*= 22). Embolization of tumor feeders was performed in 51 different segmental vessels supplying the tumor (thoracic, *n* = 29; lumbar, *n* = 22). In the thoracic region, blood supply of ten vertebras on the right, six on the left, and 13 vertebrae on both sides were embolized. In the lumbar region, three vertebrae on the right, four on the left, and 15 on both sides could be occluded. There was no statistical difference between the number of unilateral thoracic and lumbar occlusions (*p*=0.1552).

### Blood loss and neurological deficits according to time between embolization and surgery

Twenty-seven patients underwent surgery within 24 h (<24 h) after embolization and 4 patients underwent surgery after 24 to 48 h (<24 h<48 h).

There was no significant difference in intraoperative blood loss (<1000 ml or > 1000 ml) between the TSM and LSM groups operated within 24 h after embolization (*p*=0.7063) or two days after (>24 h < 48 h) (*p*>9.999).

In the <24h group, 5 patients and in the >24h<48h group, 3 patients had a new neurological deficit after intervention (embolization or surgery).

Despite the relative number of patients suffering from a new neurological deficit following embolization or surgery was higher in the group >24<48h, a statistical analysis was avoided due to the small sample size (Table 5).

**Table 5:** Blood loss and neurological deficits in relation to time between embolization and surgery

Time embolization-surgery	Blood loss		New neurology after embolization	New neurology after surgery	New neurology, after embolization direct surgery
	<1000 ml	>1000 ml			
< 24 h					
TSM (15)	10	5	1	2	1
LSM (12)	7	5	0	1	0
>24 < 48 h					
TSM (2)	1	1	1	0	0
LSM (2)	1	1	1	1	0

The table shows the classification of TSM and LSM patients in terms of time between embolization and surgery (<24 h, >24 < 28 h) and intraoperative blood loss (<1000 ml, >1000 ml). Furthermore, the rate of new neurological deficit after embolization, after surgery, or if surgery was performed immediately after embolization was evaluated in the TSM and LSM groups

The relative amount of new neurological deficits following embolization was higher in the >24<48h group: 24h: *n*%= 3,7 vs. >24<48h, *n*%= 50

The relative amount of new neurological deficits following surgery was higher in the >24<48h group: 24h: *n*%= 11 vs. >24<48h, *n*%= 25

### Chemotherapy and surgical invasiveness

Furthermore, sub-analysis of all patients was performed regarding previous or ongoing chemotherapy. Nine patients received systemic therapy with either a tyrosine kinase inhibitor (*n*=6), a monoclonal antibody (*n*=2), or an immunotherapy agent (*n*=1). Patients were also allocated to the thoracic spinal metastasis (TSM+ch) and lumbar spinal metastasis (LSM+ch) groups. Chemotherapy had no effect on ΔHb preoperatively or postoperatively between LSM and TSM within the groups (Table 2, *p*>0.05). Admitted blood units did not differ between patients who received chemotherapy (3/9) and those who did not (8/22; *p*= >0.9999) (Table 2).

There was no significant difference between the TSM (SII = 108) and LSM (SII = 83) groups (*p*=0.3925). The SII had no statistically significant effect on blood loss (<1000 ml: SII=110 points; >1000 ml: SII=77 points, *p*= 0.3622). In the group with < 1000 ml blood loss intraoperatively, the total SII was 110 points. In the group with > 1000 ml blood loss intraoperatively, the SII was 77 without a statistical significance (*p* = 0.3622).

## Discussion

Arterial embolization of the tumor-feeding vessels should be a standard procedure prior to surgery for RCC spinal metastasis due to their high vascularity [15]. Primarily, dorsal surgical procedures are performed with stabilization and decompression because of their lower perioperative morbidity compared to ventral procedures [16]. All included patients underwent dorsal stabilization. In only one patient, decompression was not performed because the metastasis was located directly in the vertebral body without risk to neurological structures.

Due to differences in the blood supply in the thoracic and lumbar regions, we reviewed patients with thoracic and lumbar metastases separately with regard to neurological deficits and blood loss.

The ΔHb was higher in the TSM group than in the LSM group as well as in the separate analysis of systemically treated patients, but without significance. After embolization, new neurological deficits occurred in two TSM patients and one LSM patient. Postoperatively, neurology occurred in both groups (3xTSM, 2xLSM).

The increased rate of neurological deficits after embolization and surgery in the thoracic spine (*n*=5) in contrast to interventions in the lumbar spine (*n*=3) can be explained by the different blood supply with few collaterals in this area.

To date, no comparison of a single hypervascular tumor entity in separate regions of the spine has been performed in previous studies with respect to intraoperative blood loss and neurological deficits.

Neurological problems occurred in three patients after embolization (presurgery). Of these, sensory deficits occurred in two patients and motor deficits occurred in one patient. Overall, we had a major complication rate of 9.6% with respect to neurological deficits, which is similar to previous reports.

Many studies reported no or only minor complications after embolization, not reaching 10% of treated patients [9, 15, 17, 18]. Jackson et al. showed that in their study of 47 patients who underwent embolization prior to surgical treatment of spinal metastasis for renal carcinoma, in 8.5% of cases, major complications occurred (permanent paraplegia, quadriparesis with improvement, and aortic dissection) [19].

In a study regarding complications after 110 embolization in spinal metastases with different entities (including 6 patients with RCC), Tang et al. found a major complication rate of 3.2% (severe hypertension, transient visual field deficit, cerebral embolism, cord ischemia). All of described complications occurred during or shortly after the procedure [8]. In two patients after embolization for spinal metastases, lower extremity paresis was noted after intervention in the Kobayashi et al. study, with no improvement despite immediate surgery with decompression. The time between embolization and onset of neurology was 2 h in one case and 6 h in the second case [20]. Thus, it is mandatory that the neurological status should be assessed after embolization and before subsequent spine surgery and documented in the patient’s file.

In the literature, surgery is recommended close to the time after embolization. The timing in the studies varied between immediate, within 24 or 48 h, or up to 72 h. Significant differences in intraoperative blood loss depending on the timing of embolization are found in only a few studies.

No difference in terms of blood loss between “immediate” and “delayed (<48 h)” groups after embolization was observed in the study by Quraishi et al. in metastatic RCC. However, different surgical methods (decompression only, decompression and stabilization, anterior only, anterior-posterior approach) were utilized in the two groups and were not equally distributed. Similar to Quraishi et al., in the present study, the subgroup sample size investigating the appropriate time between embolization and surgery is small, and thus, the results are limited. Nonetheless, an increase of the sample size is not possible due to the retrospective nature of the study. In contrast to our study, no neurological complications after embolization were observed in the study of Quraishi et al. [7].

Kato et al. recommended surgery as early as possible after embolization. In their study, they showed that intraoperative blood loss and the donation of blood units on the same day were significantly lower than on the day following embolization. It remains unclear whether the surgeries were performed by the same surgeon with the same experience [12]. In addition, the blood loss varied between both groups despite similar operation times and same operation procedure (laminectomy and two upper vertebra stabilization). It also remains to be determined whether relevant neovascularization can occur within 24 h after embolization.

Tang et al. investigated the complication rate after 110 embolizations in a population with mixed tumor entities. There was no difference in intraoperative blood loss between the group with surgery on the same day of embolization and the group with embolization the next day. However, complications after embolization occurred up to 6 h later. The recommendation in this study was to perform surgery the day after embolization [8]. Systemic therapy is the standard treatment in addition to nephrectomy for RCC. Thyrosine kinase inhibitors, immune checkpoint inhibitors, and mTOR inhibitors were used. Thyrosine kinase inhibitors, in particular, have anti-angiogenic effects [21]. In 9 of the included patients, systemic therapy was initiated by the urologist before hospital admission. A significant difference in intraoperative blood loss could not be detected in the comparison between systemically treated and non-systemically treated patients.

Overall, RCC responds poorly to chemotherapy, and in particular, higher stages have a significant relapse rate. One explanation for the missing reduction in intraoperative blood loss in this group is that systemic therapy does not have a significant therapeutic effect in patients with metastases and a progressive disease course. SII was slightly higher in the TSM (108 points) group than in the LSM (83 points) group. This is due to the fact that in the thoracic spine, 2 adjacent vertebral bodies are usually included in the stabilization. In the lumbar spine, only one adjacent vertebral body is often instrumented if stability permits. In total, only four patients (12.9%) underwent dorsoventral surgery (TSM, *n*=2; LSM, *n*=2). The goal of dorsoventral surgery in our patients was curative therapy. This high surgical effort is justifiable in cases where only a single metastasis is present and the tumor has a good prognosis [22].

One strength of our study is the homogeneous patient group. We included only patients with histologically proven metastases of RCC and embolization prior to surgical therapy. Few studies have previously performed a dedicated evaluation of this patient group. Frequently, intraoperative blood loss after embolization of a spinal metastasis was evaluated mostly in a mixed population of different tumor entities. In addition, the number of 31 included patients should be emphasized in comparison to the literature, with mean patient numbers of 26.4 [5, 12, 15, 19, 23–25]. Significant differences in terms of descriptive data between the TSM and LSM groups do not exist, which is a strength of this comparative study. A dedicated evaluation according to the location of spinal metastases in RCC has not yet been performed because of the small study population in published studies.

One limitation of this study is its retrospective study design. We also did not perform a precise analysis of the size of the treated metastases and the degree of vascularization. We also did not explicitly analyze the time of onset of new neurological deficits after embolization. We only evaluated the records to determine if any new neurological deficits occurred during the period between embolization and surgery.

Furthermore, the differences in size of the metastasis as well as individual parameters like body mass index, height, and weight as well as tumor size and the resulting operation time might affect the blood loss and were not considered here. For this purpose, a matched pair study would be required.

Nevertheless, our results provide guidance for practicing spine surgeons in planning and performing surgery for metastases of RCC. In particular, the logistic challenges in the hospital in terms of planning of inpatient admission, embolization, and surgery for these patients is a great challenge for the treating physicians each time.

## Conclusions

Embolization is a standard procedure prior to surgery for hypervascular spinal metastases, regardless of the region and complexity of the surgical procedure. Embolization is also possible up to 48 h before surgery, without any risk of intraoperative blood loss. We recommend surgery the day after embolization to reliably detect neurological complications arising after this procedure. After embolization, neurological monitoring of the patient is mandatory to detect serious post-interventional complications and to initiate appropriate therapeutic steps.

## Data Availability

All relevant data generated or analyzed during the current study are presented in the paper. The detailed datasets used during the current study are available from the corresponding author upon reasonable request.

## Abbreviations

TSM: Thoracic spinal metastasis
TSM+ch: Thoracic spinal metastasis and previous/ongoing chemotherapy
LSM: Lumbar spinal metastasis
LSM+ch: Lumbar spinal metastasis and previous/ongoing chemotherapy
RCC: Renal cell carcinoma
